# NK-4 exerts selective regulatory effects on the activation and function of allergy-related Th2 cells

**DOI:** 10.1371/journal.pone.0199666

**Published:** 2018-06-22

**Authors:** Keizo Kohno, Satomi Koya-Miyata, Akira Harashima, Toshio Ariyasu, Shimpei Ushio

**Affiliations:** R&D Division, Hayashibara Co., Ltd., Okayama, Japan; Mie University Graduate School of Medicine, JAPAN

## Abstract

NK-4 is the main component of the antiallergic drug Lumin, which has been in popular usage since the early 1950s. In this study, we examined whether NK-4 exerts a regulatory effect on the activation and effector function of Th2 cells. NK-4 inhibited IL-4 production by anti-CD3ε mAb-stimulated BALB/c mouse spleen cells, whereas NK-4 had little effect on IFN-γ production. IL-4 and IL-5 secretion by anti-CD3ε mAb- or antigen-stimulated Th2 cells (D10.G4.1) was abrogated by NK-4 without affecting cell numbers, whereas IFN-γ secretion by activated Th1 cells was unchanged. Mechanistic analysis revealed that NK-4 inhibited mRNA expression of the Th2-associated transcription factors GATA-3 and NFATc1 in anti-CD3ε mAb-stimulated D10.G4.1 cells. Regarding the regulation of Th2 cell effector functions, NK-4 inhibited the secretion of eotaxin and thymus and activation-regulated chemokine (TARC) by normal human dermal fibroblasts in response to IL-4 and/or TNF-α. NK-4 achieved TARC attenuation comparable to what is observed with suplatast tosilate, an antiallergic drug that selectively inhibits Th2 cytokine production, at 14-fold lower concentrations of suplatast tosilate. Dexamethasone increased TARC production by 2.2- to 2.6-fold of control cultures. NK-4 successfully inhibited the STAT6 signaling pathway, suggesting a potential mechanism for down-regulating chemokines expression. In addition, NK-4 abrogated IL-4-driven modulation of cytokine production profile in human monocytic THP-1 cells from proinflammatory to anti-inflammatory response, as seen in the inverted ratio of TNF-α to IL-10 produced in response to LPS. These results suggest that NK-4 could prevent IL-4-driven polarization to alternatively activated macrophages, which are proposed to have pathogenic roles in allergic asthma. The importance of Th2 cytokines and chemokines in the development and progression of type 2 inflammatory disorders has been highlighted by recent advance in our understanding the immunological mechanism underlying allergic disease. Our results support the use of NK-4 as a reasonable therapeutic option to alleviate Th2-mediated allergic inflammation.

## Introduction

CD4^+^ effector T helper (Th) cells play central roles in host defense against a range of invading pathogens. Since the discovery of Th1 and Th2 cells in 1986 [1], several lineages of CD4^+^ Th cells have been identified [2]. Th1 cells that secrete IFN-γ upon antigenic stimulation have a critical role in the eradication of intracellular pathogens, since IFN-γ produced by Th1 cells is a key factor in the elimination of intracellular pathogen by increasing the level of cellular reactive oxygen species (ROS) [3].

In helminth infections, the host immune system promotes Th2 commitment by naïve Th cells. It is now clear that proteases derived from helminths initiate this process [4]. Helminth-specific Th2 cells, in turn, stimulate B cells to switch from IgM to IgE synthesis. Th2 cells and IgE-bound mast cells are activated by helminth-derived antigens and promote the accumulation of eosinophils and basophils through the secretion of Th2 cytokines and chemokines. IgE promotes parasite expulsion from the gut and regulates mast cell responses against helminths [5]. Eosinophils are well-known to accumulate around helminths and to release ROS and toxic granular proteins upon stimulation.

Thus, although Th2 cells play an essential function in the host defense against helminth invasion, Th2 cells orchestrate allergic inflammatory responses such as asthma and atopic dermatitis as the result of exposure of the hosts to exogenous allergic molecules. As in the case of helminth infection, Th2 cells induce IgE production by B cells. Mast cells and basophils are activated by IgE binding to their high affinity IgE receptors. Upon reexposure to allergen these cells degranulate and release mediators that induce bronchoconstriction and airway hyperresponsiveness. Eosinophils are also recruited by the eosinophil chemoattractant eotaxin in the lungs of asthmatic patients, where they are involved in airway hyperresponsiveness and remodeling [6]. Eotaxin is secreted from lung epithelial cells, fibroblasts and smooth muscle cells in response to IL-4, IL-13 and TNF-α that are produced by activated mast cells and Th2 cells [6, 7]. Thus, allergen-induced Th2 cells play essential roles in the development of allergic inflammatory diseases. However, therapeutic strategies for allergic inflammatory diseases by directly regulating the effector function of Th2 cells remain limited, whereas symptomatic treatments using antihistamine drugs and corticosteroids have been well established.

NK-4 is a divalent cationic pentamethine trinuclear cyanine dye that contains three quinolinium rings, N-ethyl side chains and two iodine anions. NK-4 inhibited IgE production and IgE-mediated passive cutaneous anaphylaxis *in vivo* [8]. We observed that oral administration of NK-4 (1 mg/kg) for 3 days to C57BL/6N mice increased the population of invariant NKT (iNKT) cells that secreted higher levels of IFN-γ upon stimulation with α-galactosylceramide, when compared to iNKT cells from vehicle-administered mice [9]. Grela F et al. reported that IFN-γ-producing iNKT cells alleviated allergic inflammation [10]. These results suggest that NK-4 has a potential application in the treatment of allergic diseases. However, it remains unclear that NK-4 exhibits antiallergic actions by modulating the activation and effector function of Th2 cells that play major roles in the development of allergic inflammatory responses.

In this study, we investigated whether NK-4 exerts a regulatory effect on cytokine secretion by Th1 or Th2 cells as well as primary splenic T cells. Since Th2 cytokine-selective down-regulation was observed with NK-4, we next examined whether NK-4 modulates the effector function of IL-4 and TNF-α, both of which are produced by activated Th2 cells, mast cells and basophils in the course of allergic inflammatory responses [6, 7]. For this purpose, we investigated IL-4- and/or TNF-α-induced Th2 chemokine secretion by dermal fibroblasts and IL-4-driven modulation of cytokine production profile in human monocytic THP-1 cells from proinflammatory to anti-inflammatory response. We demonstrated that NK-4 exerts a selective regulatory effect on the activation of Th2 cells and on the secretion of Th2-associated chemokines by fibroblasts induced by Th2 cytokines.

## Materials and methods

### Reagents

**Fig 1:**
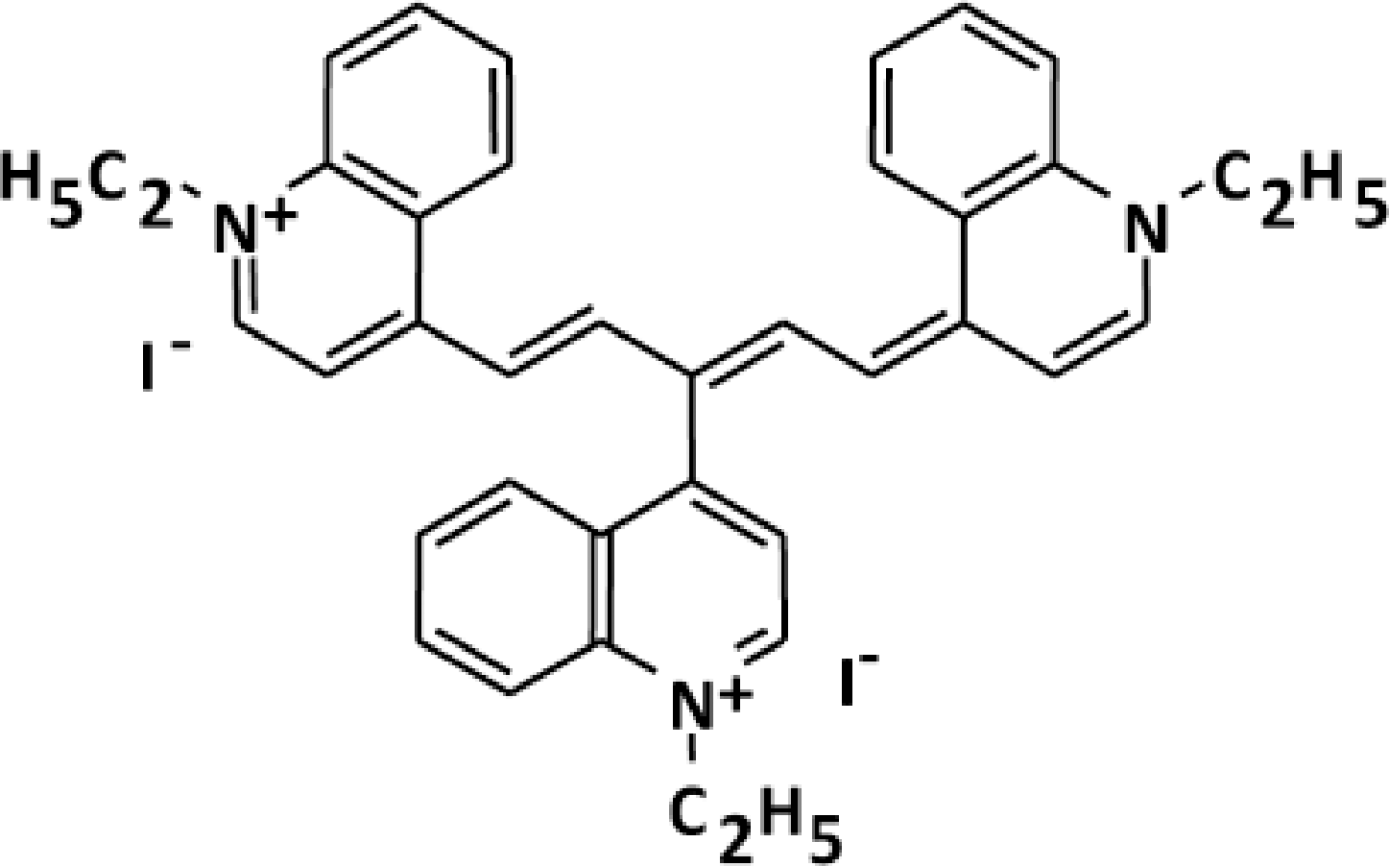
Chemical structure of NK-4.

NK-4, 1-ethyl-4-[(1Z,3E,5E)-1-(1-ethylquinolin-1-ium-4-yl)-5-(1-ethylquinolin-4-ylidene)penta-1,3-dien-3-yl]quinolin-1-ium;iodide, was synthesized at Fine Chemicals & Wellness Products Department, Hayashibara Co., Ltd. (Okayama Japan) (Fig 1). NK-4 is the common name of cryptocyanine O.A.1 (PubChem CID: 5489539). A stock solution of 10 mM NK-4 was prepared with DMSO and stored at room temperature with protection from light. Final concentrations of DMSO were 0.15% or less, and did not affect the results of the experiments. Dexamethasone, FK-506 and suplatast tosilate (IPD-1151T) were purchased from Sigma-Aldrich (St. Louis, MO). Recombinant human IL-4 was purchased from R&D Systems (Minneapolis, MN). Human TNF-α was prepared in our laboratory.

### Mice

BALB/c and C57BL/6 female mice, aged 7–8 weeks, were purchased from Charles River Japan (Kanagawa, Japan). Mice were fed rodent chow (Oriental Yeast, Tokyo, Japan) and water *ad libitum*. All animal experiments were approved by the Animal Care and Use Committee of R&D Center of Hayashibara Co., LTD. (Approved Number: hb1608-03). Mice were euthanized by cervical dislocation.

### T cell clones

OVA-specific Th1 clone #4 was established from BALB/c mice in our laboratory [11], and were maintained by repeated stimulation with OVA and mitomycin C (MMC)-treated BALB/c spleen cells and subsequent culture in medium containing 10 U/ml human IL-2. An ovalbumin-specific Th2 clone, D10.G4.1, was provided by Dr. H. Nariuchi, Institute of Medical Science, University of Tokyo, and was maintained by repeated stimulation with MMC-treated C57BL/6 spleen cells, as described previously [11]. All cell cultures used complete medium comprised of RPMI 1640 supplemented with 10% FCS (Life Technologies, Grand Island, NY), 50 μM 2-ME, 60 μg/ml penicillin, and 50 μg/ml streptomycin. These T cell clones were used for experiments after culture for at least 2 weeks following the last antigen stimulation.

### Stimulation of splenic T cells and T cell clones

BALB/c moue spleen cells (1.2 x 10^6^ cells/well) were stimulated with soluble 5 μg/ml anti-CD3ε mAb (145-2C11; Cedarlane Laboratories, Ontario, Canada) in the presence or absence of varying concentrations of NK-4 for 48 h at 37°C in 96-well flat-bottom plate.

Th1 clone #4 and Th2 clone D10.G4.1 cells (2.5 x 10^4^ cells/well) were stimulated with immobilized anti-CD3ε mAb (8 μg/ml) in the presence or absence of varying concentrations of NK-4 for 48 h at 37°C in 96-well plates. For antigenic stimulation, Th1 clone #4 cells (2.5 x 10^4^ cells/well) were stimulated with OVA (200 μg/ml) and 50 μg/ml MMC-treated BALB/c mouse spleen cells (1.2 x 10^6^ cells/well) as antigen presenting cells in the presence or absence of NK-4 for 48 h at 37°C in 96-well plates. D10.G4.1 cells (2.5 x 10^4^ cells/well) were stimulated allogenically with MMC-treated C57BL/6 mouse spleen cells (1.2 x 10^6^ cells/well) [11]. Concentrations of IFN-γ and IL-4 in culture supernatants were measured by ELISA. Cell numbers were determined by cell counting kit-8 (Wako Pure Chemical, Osaka, Japan). Briefly, 10 μl WST-8 reagent, a water-soluble tetrazolium salt, was added to each microplate well for the last 2 to 3 h of the incubation period. Optical density was measured at 450 nm.

### Cytokine assays

Culture supernatant cytokines (mouse IL-4, IL-5 and IFN-γ) were measured by two-site sandwich ELISA. The Abs for coating the plates and the biotinylated secondary mAbs were as follows: for IL-4, rat mAb anti-mouse IL-4 (BVD4-1D11, BD Biosciences, San Diego, CA) and biotinylated mAb anti-mouse IL-4 (BVD6-24G2, BD Biosciences); for IL-5, rat mAb anti-mouse/human IL-5 (TRFK5, BD Biosciences) and biotinylated mAb anti-mouse IL-5 (TRFK4, BD Biosciences); for IFN-γ, polyclonal rabbit anti-mouse IFN-γ Ab (prepared in our laboratory) and biotinylated rat mAb anti-mouse IFN-γ (XMG1.2, BD Pharmingen). HRPO-conjugated Streptavidin was purchased from Zymed (San Francisco, CA). Levels of human TNF-α were determined by an ELISA system that was developed in our laboratory. Levels of human IL-10 were determined with human IL-10 ELISA Ready-SET-Go kit (Thermo Fisher Scientific, Waltham, MA). Eotaxin and thymus and activation-regulated chemokine (TARC) were measured with human CCL11/Eotaxin DuoSet ELISA Development Systems (R&D Systems, DY320) and human CCL17/TARC DuoSet ELISA Development Systems (R&D Systems, DY364), respectively.

### RNA extraction and quantitative real-time polymerase chain reaction (RT-PCR)

Briefly, D10.G4.1 cells (2 x 10^6^ cells/well) were dispensed into anti-CD3 mAb coated 6-well plates, with or without varying concentrations of NK-4, and incubated for 6 h at 37°C. Total RNA was extracted from D10.G4.1 cells using an RNeasy Mini kit (QIAGEN, Tokyo, Japan) and DNase (QIAGEN) according to the manufacturer’s instructions. Subsequently, first-strand cDNA was synthesized using SuperScript™ VILO™ Master Mix (Invitrogen, Carlsbad, CA). All PCR primer sequences are shown in Table 1. Synthesized cDNA was mixed with SYBR Green Master Mix (Roche, Mannheim, Germany) and different sets of gene-specific primers. Real-time PCR was performed using a LightCycler 480 system (Roche). For quantitative purposes, the expression of each gene was normalized to the house keeping gene 18S rRNA.

**Table 1:** Primers used for quantitative real-time PCR analysis.

	Primer sequences (5’ → 3’)			
Gene name	Forward	Reverse	Amplicon	Gene Bank
			Size	Accession No
18S rRNA	GGACACGGACAGGATTGACA	ACCCACGGAATCGAGAAAGA	50	Reference^a^
Mus Gata3	TTATCAAGCCCAAGCGAAG	TGGTGGTGGTCTGACAGTTC	75	NM_008091.3
Mus c-Maf	CCTTCCTCTCCCGAATTTTT	CCACGGAGCATTTAACAAGG	73	NM_001025577.2
Mus Nfatc1	TCCAAAGTCATTTTCGTGGA	CTTTGCTTCCATCTCCCAGA	63	NM_016791.4

^a^Reference; Specific primers for PCR analysis of 18s rRNA were identical to those described by Steen E et al. (11)

### Th2 chemokine production by normal human dermal fibroblasts (NHDF)

NHDF derived from neonatal foreskin (KF-4009, KURABO, Osaka, Japan) were cultured in Dulbecco’s minimum essential medium (D-MEM) containing 10% FCS until confluent in 96-well plates. Culture medium was replaced with D-MEM containing 0.1% bovine serum albumin (Biocompare, San Francisco), and cells were then stimulated for 48 hr with varying concentrations of IL-4 and/or TNF-α in the presence or absence of NK-4. For induction of TARC, NHDF were stimulated with a combination of 10 ng/ml IL-4 and 5 ng/ml TNF-α in the presence or absence of NK-4, dexamethasone or FK-506. In experiments comparing NK-4 with suplatast tosilate, NHDF were exposed to test articles for 15 min before stimulation with IL-4 and TNF-α, since suplatast tosilate had been pre-incubated for 10 min before stimulation in several *in vitro* studies [12–14]. After the incubation period, levels of eotaxin and TARC in the culture supernatants were measured by specific ELISA. Cell numbers were determined by cell counting kit-8.

### Modulation of cytokine production profile in THP-1 cells by IL-4

THP-1 cells (JCRB0112, Japanese Collection of Research Bioresources Cell Bank, Osaka, Japan), a human monocytic cell line, were cultured with IL-4 for 3 days at 37°C in the presence or absence of varying concentrations of NK-4 in RPMI1640 medium containing 1% FCS in 12-well culture plates. After the incubation period, cells were recovered and washed twice with the same medium. Cells were then suspended in fresh RPMI1640 medium containing 10% FCS and viable cell numbers were determined by trypan blue exclusion. Recovered THP-1 cells (1.2 x 10^5^ cells/well) were stimulated overnight with 5 μg/ml LPS in 96-well plates. Levels of TNF-α and IL-10 in the culture supernatants were determined by specific ELISA. In some experiments, viable cell numbers were assessed by trypan blue exclusion after overnight stimulation with LPS.

### Western immunoblotting analysis of signal transducer and activator of transcription factor 6 (STAT6)

Western blotting was performed using whole cell extracts from NHDF cells treated with 10 ng/ml IL-4 and 5 ng/ml TNF-α in the presence or absence of varying concentrations of NK-4. Membranes were probed with a 1:1000 dilution of anti-phospho-STAT6 (Tyr641) rabbit mAb (C11A12; Cell Signaling Technology, Danvers, MA). Specific bands were detected using an ECL^™^ Prime Western Blotting System (Immobilon Western Chemiluminescent HRP substrate; GE Healthcare, UK). After treatment with a reprobing solution (Restore Western Blot Stripping Buffer; Pierce Biotechnology, Rockford, IL) for 15 min at room temperature, the membrane was used for secondary detection with a 1:1000 dilution of anti-STAT6 rabbit pAb (SC-621; Santa Cruz Biotechnology, Santa Cruz, CA). Band density was measured using Image J software.

### Statistical analysis

The data were analyzed by one-way analysis of variance followed by Dunnett’s multiple-comparison test. *P* values ≤ 0.05 were considered statistically significant.

## Results

### NK-4 down-regulates Th2 cytokine production by established Th2 cells as well as by primary splenic T cells

**Fig 2:**
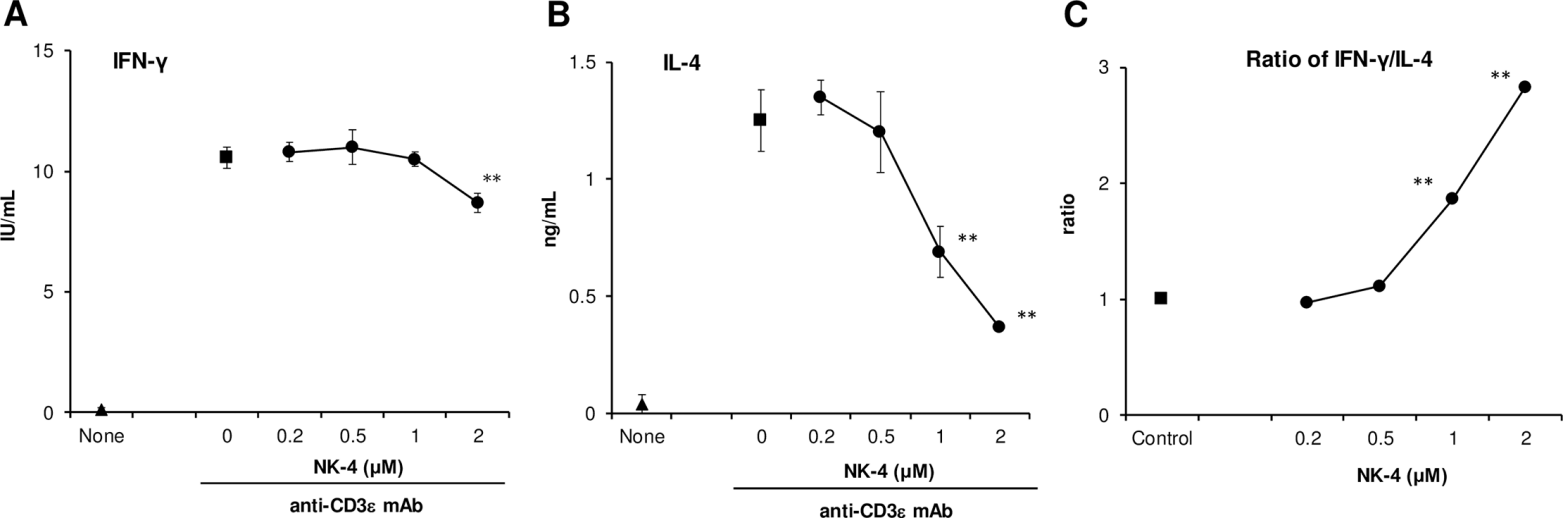
NK-4 selectively down-regulates IL-4 production by primary splenic T cells in response to anti-CD3ε mAb. BALB/c moue spleen cells (1.2 x 10^6^ cells/well) were stimulated with 5 μg/ml anti-CD3ε mAb in the presence or absence (control) of varying concentrations of NK-4 for 48 h at 37°C in 96-well plates. Concentrations of IFN-γ (A) and IL-4 (B) in culture supernatants were measured by ELISA. The ratios of IFN-γ to IL-4 are shown (C). Results are the means ± S.D. of triplicate cultures. Results are representative of two independent experiments with similar results. **p < 0.01 compared with control cultures.

We examined whether NK-4 has a regulatory effect on Th1/Th2 cytokine production by primary T cells. For this purpose, mouse spleen cells were stimulated with soluble anti-CD3ε mAb for 48 h, and the levels of IL-4 and IFN-γ in the culture supernatants were measured. As shown in Fig 2A and 2B, anti-CD3ε mAb induced BALB/c mouse splenic T cells to produce 10.6 ± 0.4 IU/ml (means ± S.D., n = 3) IFN-γ and 1.25 ± 0.13 ng/ml (means ± S.D., n = 3) IL-4 in the culture supernatants. NK-4 dose-dependently inhibited IL-4 production, whereas IFN-γ production was little affected by the addition of NK-4. At 1 and 2 μM NK-4, IL-4 production was significantly inhibited 45% and 71%, respectively. The ratio of IFN-γ to IL-4 levels therefore significantly increased in an NK-4 dose-dependent manner (Fig 2C).

**Fig 3:**
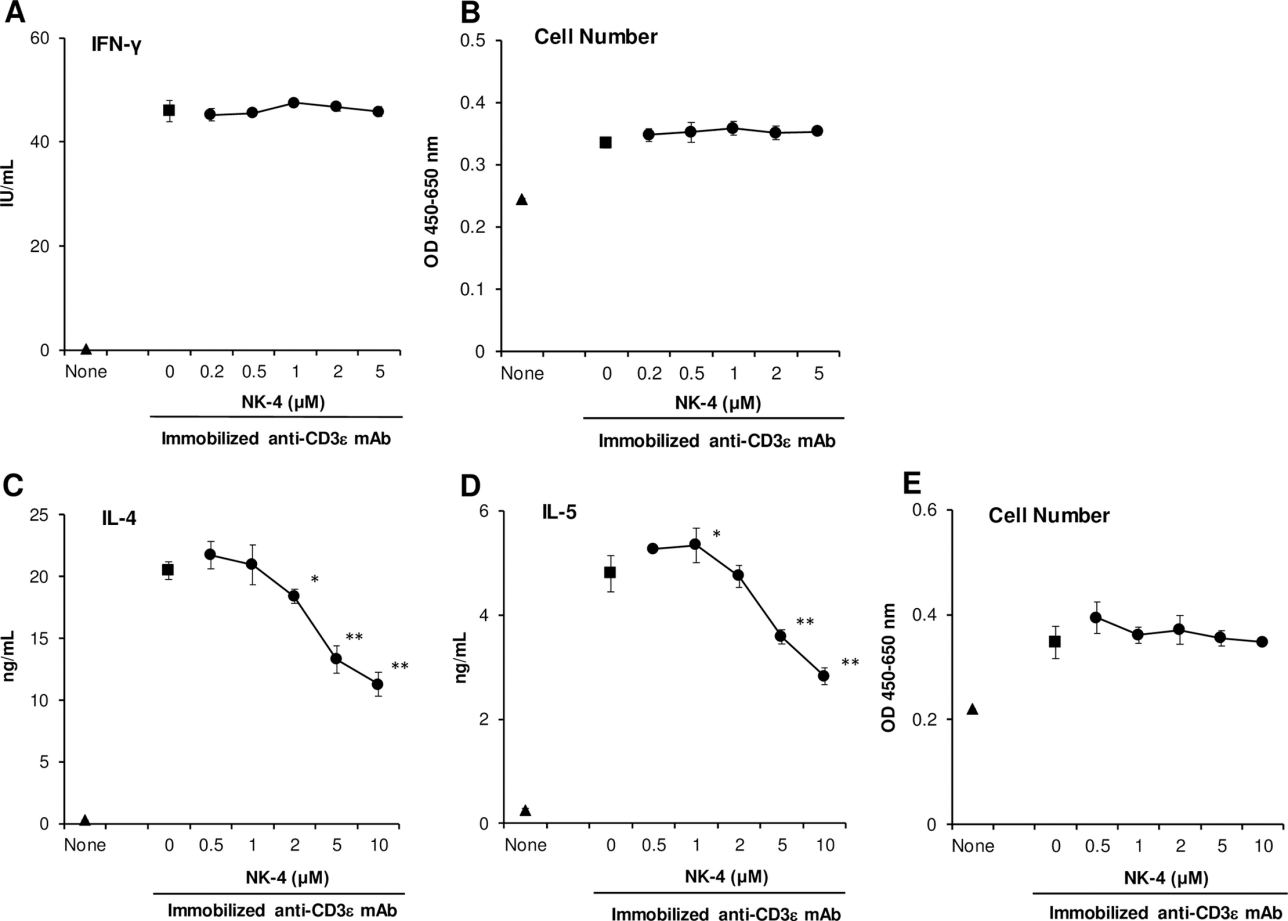
NK-4 selectively down-regulates Th2 cytokine production by anti-CD3ε mAb-stimulated established Th2 cells. Th1 clone #4 (A and B) and Th2 clone D10.G4.1 cells (C to E) (2.5 x 10^4^ cells/well) were stimulated with immobilized anti-CD3ε mAb (8 μg/ml) in the presence or absence (control) of varying concentrations of NK-4 for 48 h at 37°C in 96-well plates. Concentrations of IFN-γ (A), IL-4 (C) and IL-5 (D) in culture supernatants were measured by ELISA. Cell numbers of #4 (B) and D10.G4.1 (E) were determined by cell counting kit-8. Results are the means ± S.D. of triplicate cultures. Results are representative of three independent experiments with similar results. *p < 0.05, **p < 0.01 compared with control cultures.

To further examine whether NK-4 selectively inhibits Th2 cytokine production, established mouse Th1 and Th2 cells were stimulated with immobilized anti-CD3ε mAb for 48 h in the presence or absence of varying concentrations of NK-4. As shown in Fig 3A and 3B, NK-4 affected neither IFN-γ secretion nor the cell numbers of OVA-specific Th1 clone #4 cells. In marked contrast, NK-4 significantly and dose-dependently inhibited the secretion of both IL-4 and IL-5 by conalbumin-specific Th2 clone D10.G4.1 cells without affecting the cell numbers (Fig 3C–3E).

**Fig 4:**
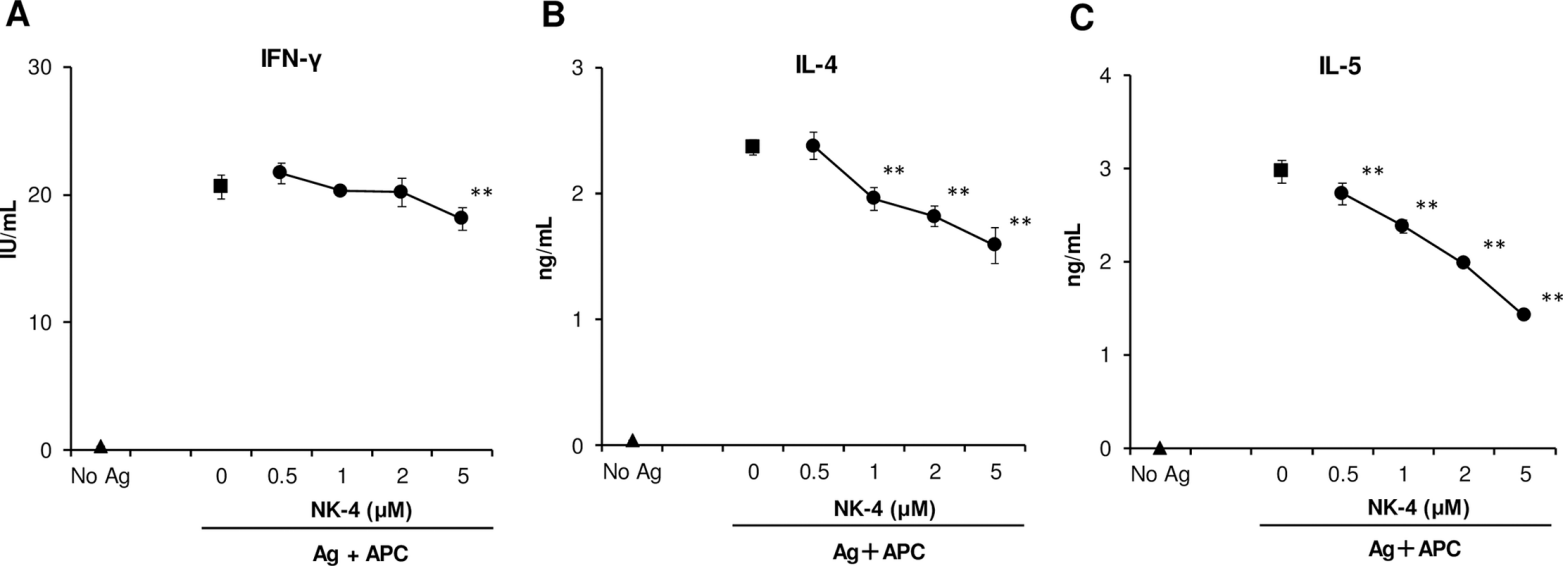
NK-4 selectively down-regulates Th2 cytokine production by antigen-stimulated established Th2 cells. Th1 clone #4 (2.5 x 10^4^ cells/well) were stimulated with OVA (200 μg/ml) and MMC-treated BALB/c mouse spleen cells (1.2 x 10^6^ cells/well) in the presence or absence (control) of varying concentrations of NK-4 for 48 h at 37°C in 96-well plates (A). D10.G4.1 cells (2.5 x 10^4^ cells/well) were stimulated with MMC-treated C57BL/6 mouse spleen cells (1.2 x 10^6^ cells/well) (B and C). Concentrations of IFN-γ (A), IL-4 (B) and IL-5 (C) in culture supernatants were measured by ELISA. Results are the means ± S.D. of triplicate cultures. Results are representative of three independent experiments with similar results. **p < 0.01 compared with control cultures.

Th2 cytokine-selective inhibition by NK-4 was also observed when Th1 and Th2 cells were stimulated under physiological conditions with specific antigen and antigen- presenting cells (Fig 4). The addition of NK-4 resulted in a dose-dependent decrease in both IL-4 and IL-5 secretion by antigen-stimulated D10.G4.1 cells, whereas IFN-γ secretion by Th1 clone #4 was not affected. At 5 μM NK-4, IL-4 and IL-5 production were inhibited 33% and 52%, respectively. These results suggest that NK-4 exhibits a down-regulatory effect on Th2 cytokine production by established Th2 cells as well as on primary T cells. It should be noted that the inhibitory effect of NK-4 on Th2 cytokine production is by direct action on Th2 cells as observed with immobilized anti-CD3ε mAb stimulation (Fig 3C and 3D).

### NK-4 suppresses mRNA expression of the Th2-associated transcription factors GATA-3 and NFATc1

**Fig 5:**
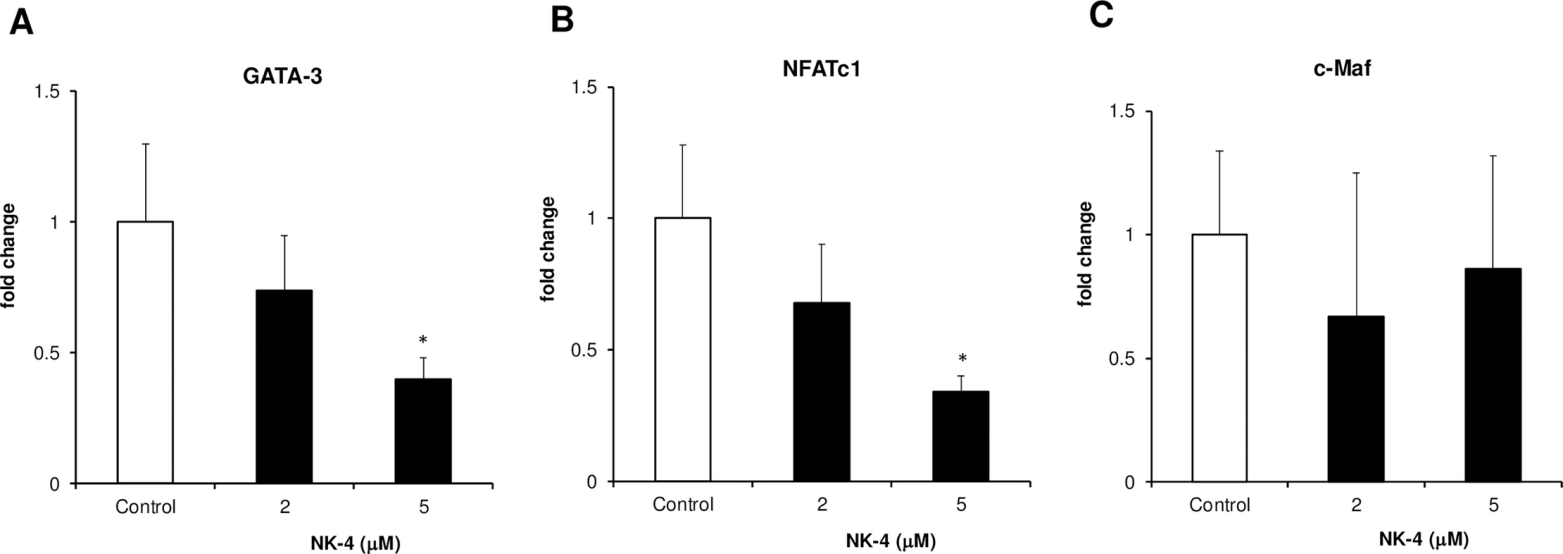
NK-4 suppresses the mRNA expression of the Th2-associated transcription factors GATA-3 and NFATc1. Total RNA was extracted from D10.G4.1 cells (2 x 10^6^ cells) stimulated with immobilized anti-CD3ε mAb (8 μg/ml) in the presence or absence (control) of NK-4 for 6 h at 37°C. First-strand cDNA was synthesized using reverse transcriptase, as described in the Materials and Methods. Expression levels of GATA-3 (A), NFATc1 (B) and c-Maf (C) were analyzed by real-time PCR. 18S rRNA expression levels were used for normalization. Results are given as changes in gene expression relative to the mean values of the control. Results are the means ± S.D. of three independent experiments. *p < 0.05, compared with control cultures.

Since NK-4 directly suppressed IL-4 and IL-5 production by anti-CD3ε mAb-stimulated D10.G4.1 cells, we speculated that NK-4 would suppress the expression of Th2-associated transcription factor(s) that are involved in Th2 cytokine mRNA expression. It is well established that GATA-3 and c-Maf are critical Th2-specific transcription factors [15]. Although its expression is not Th2-specific, NFATc1 activates IL-4 gene expression directly or in concert with c-Maf and GATA-3 [15]. We therefore examined the effect of NK-4 on the mRNA expression of these three Th2-associated transcription factors in immobilized anti-CD3ε mAb-stimulated D10.G4.1 cells using quantitative real-time PCR. As shown in Fig 5, NK-4 significantly and dose-dependently suppressed GATA-3 and NFATc1 mRNA expression, while NK-4 had no effect on c-Maf mRNA expression. At 5 μM NK-4, GATA-3 and NFATc1 mRNA expression was significantly inhibited 60% and 66%, respectively.

### NK-4 down-regulates NHDF secretion of eotaxin and TARC in response to IL-4 and/or TNF-α

Eotaxin (CCL11), a Type-2 chemokine, is a potent chemoattractant and activator of CC chemokine receptor-3 (CCR3)-expressing eosinophils that accumulate in high numbers in the lungs of asthmatic patients as well as in the lesional skin from atopic dermatitis patients [6, 16]. Eotaxin is released from epithelial and dermal fibroblasts in response to IL-4, IL-13 or TNF-α either stimulated independently or in various combinations. These cytokines are secreted from activated mast cells, basophils and Th2 cells [6, 17, 18]. We next examined the regulatory effect of NK-4 on the release of eotaxin from NHDF.

**Fig 6:**
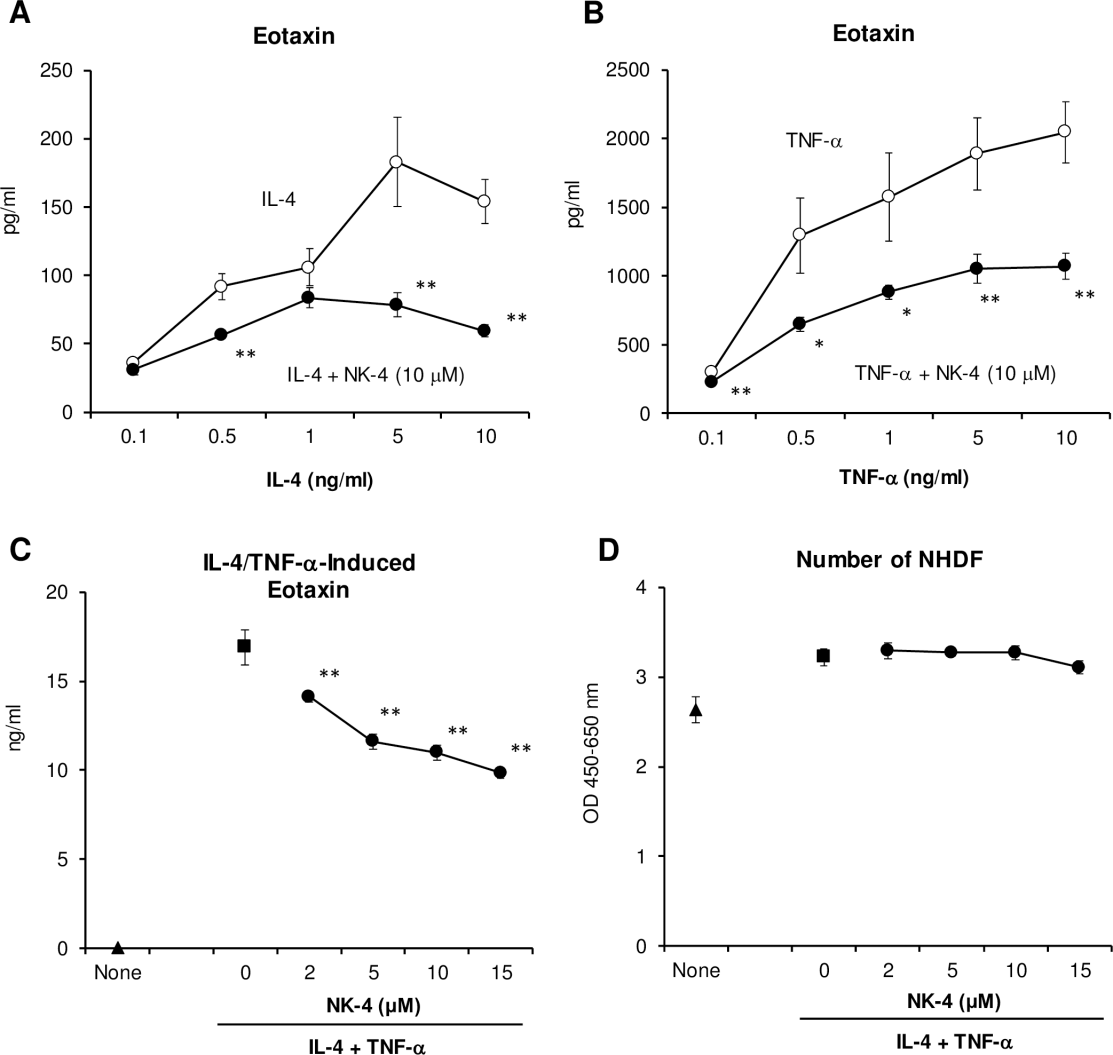
NK-4 down-regulates secretion of eotaxin by NHDF in response to IL-4 and/or TNF-α. NHDF were stimulated with varying concentrations of IL-4 (A) or TNF-α (B) in the presence or absence (control) of 10 μM NK-4 for 48 h at 37°C. The effect of NK-4 on the secretion of eotaxin from NHDF stimulated with 10 ng/ml IL-4 and 5 ng/ml TNF-α was assessed (C and D). Concentrations of eotaxin in culture supernatants were measured by ELISA (A to C). Cell numbers of NHDF were determined by cell counting kit-8 (D). Results are the means ± S.D. of triplicate cultures. Results are representative of three independent experiments with similar results. *p < 0.05, **p < 0.01 compared with control cultures.

As shown in Fig 6A and 6B, the secretion of eotaxin from NHDF was induced during a 48 h exposure to IL-4 or TNF-α, respectively, in a dose-dependent fashion. When 10 μM NK-4 was added to the culture together with graded doses of IL-4 or TNF-α, eotaxin production was significantly inhibited at all concentrations examined. NK-4 down-regulated eotaxin secretion 62% and 48% in response to 10 ng/ml IL-4 or TNF-α, respectively. In these cultures, NK-4 had no effect on the cell numbers of NHDF (data not shown). When NHDF were stimulated by combinations of 10 ng/ml IL-4 and 5 ng/ml TNF-α, eotaxin secretion was synergistically increased as reported previously [17, 18], and 16.9 ng/ml of eotaxin was detected in the culture supernatants (Fig 6C). The addition of NK-4 significantly and dose-dependently inhibited the secretion of eotaxin by NHDF without affecting the cell numbers (Fig 6C and 6D). Eotaxin secretion was decreased to 58% of control culture at 15 μM NK-4.

**Fig 7:**
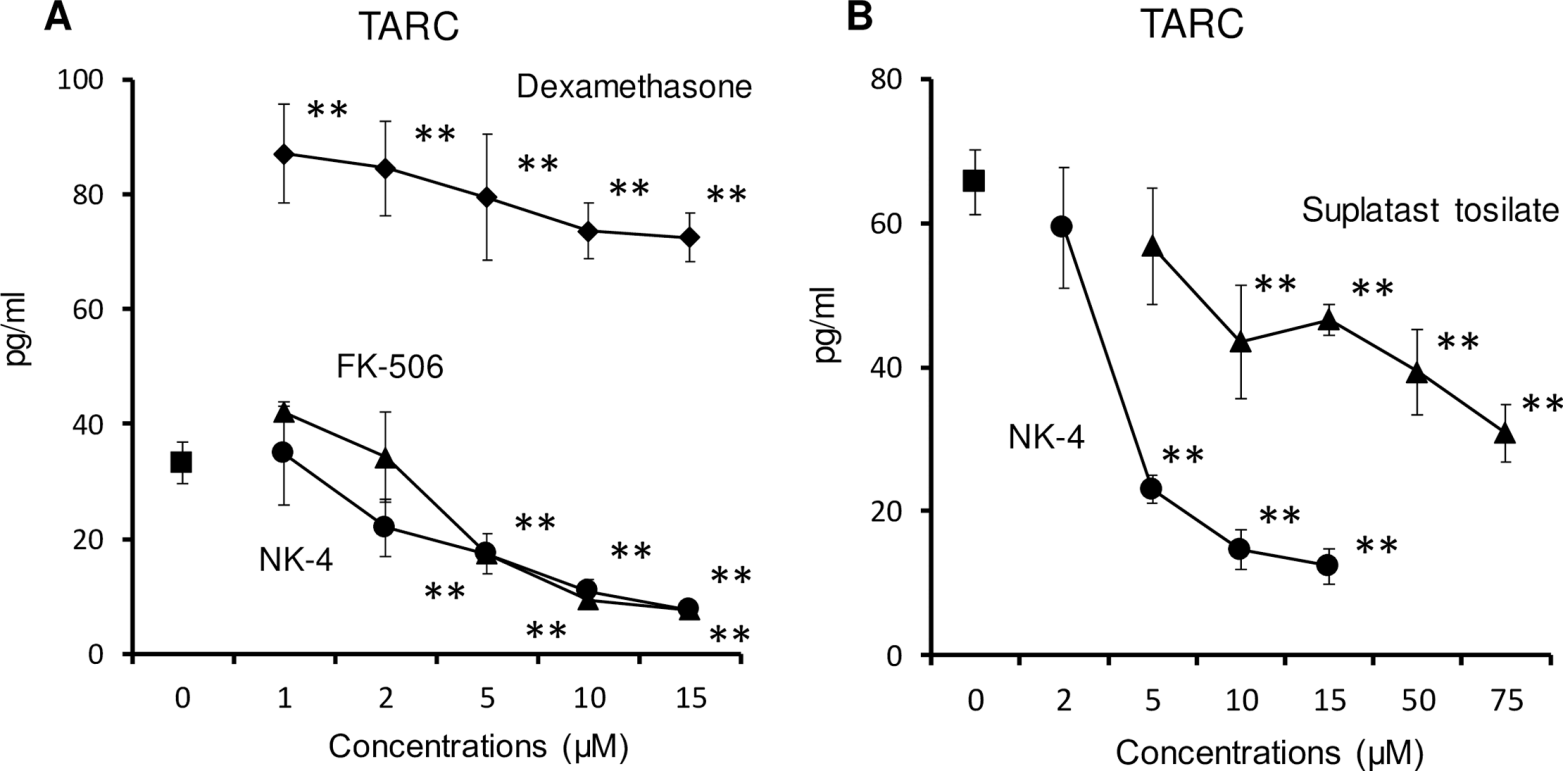
NK-4 down-regulates secretion of TARC by NHDF stimulated with both IL-4 and TNF-α. NHDF were stimulated with 10 ng/ml IL-4 and 5 ng/ml TNF-α in the presence or absence (control) of varying concentrations of NK-4, dexamethasone or FK-506 (A) for 48 h at 37°C. In experiments comparing NK-4 with suplatast tosilate (B), NHDF were exposed to test articles for 15 min before stimulation with IL-4 and TNF-α. TARC levels in the cultures of NHDF without stimulation were below detectable limits. Results are the means ± S.D. of triplicate cultures. Results are representative of three independent experiments with similar results. **p < 0.01 compared with control cultures.

TARC (CCL17) is a Type-2 chemokine. CCR4-expressing Th2 cells are recruited by TARC to sites of allergic inflammation. Since TARC is produced by corneal and dermal fibroblasts after stimulation with TNF-α and IL-4 or IL-13 [19], we next assessed the effect of NK-4 on the secretion of TARC by NHDF. As reported previously [19, 20], TNF-α or IL-4 alone did not induce NHDF to produce TARC (data not shown). However, when NHDF were stimulated with both 10 ng/ml IL-4 and 5 ng/ml TNFα, significant amounts of TARC were detected in the culture supernatants (Fig 7A). The addition of NK-4 significantly and dose-dependently inhibited TARC production by NHDF stimulated with IL-4 and TNF-α. At 15 μM NK-4, the levels of TARC in the culture supernatants were decreased to 23% of control cultures. The ability of NK-4 to inhibit TARC secretion from NHDF was compared with that of FK-506 and dexamethasone (Fig 7A), both of which are frequently used as immunosuppressive drugs. FK-506 also significantly inhibited TARC secretion at concentrations over 5 μM. However, dexamethasone did not inhibit TARC secretion, even though the dose was increased to 15 μM. Instead, dexamethasone increased TARC secretion by 2.2- to 2.6-fold of control cultures. These results suggest that NK-4 may attenuate the infiltration of eosinophils and Th2 cells into lung tissue during the effector phase of allergic airway diseases by down-regulating Th2 chemokine secretion.

Suplatast tosilate (IPD-1151T) selectively inhibits Th2 cytokine production and has been used as an antiallergic drug [21]. Suplatast tosilate also inhibits IL-4-induced eotaxin production by NHDF [22]. We then compared the efficacy of the two drugs, NK-4 and suplatast tosilate, for down-regulating TARC production by NHDF stimulated with IL-4 and TNF-α. As shown in Fig 7B, both NK-4 and suplatast tosilate significantly and dose-dependently inhibited the secretion of TARC by NHDF. However, NK-4 was superior to suplatast tosilate in attenuating TARC production (IC_50_: 4.0 ± 0.7 μM for NK-4, 55 ± 10 μM for suplatast tosilate, means ± S.D., n = 3).

### NK-4 inhibits IL-4-induced alteration of cytokine expression profile in THP-1 cells

**Fig 8:**
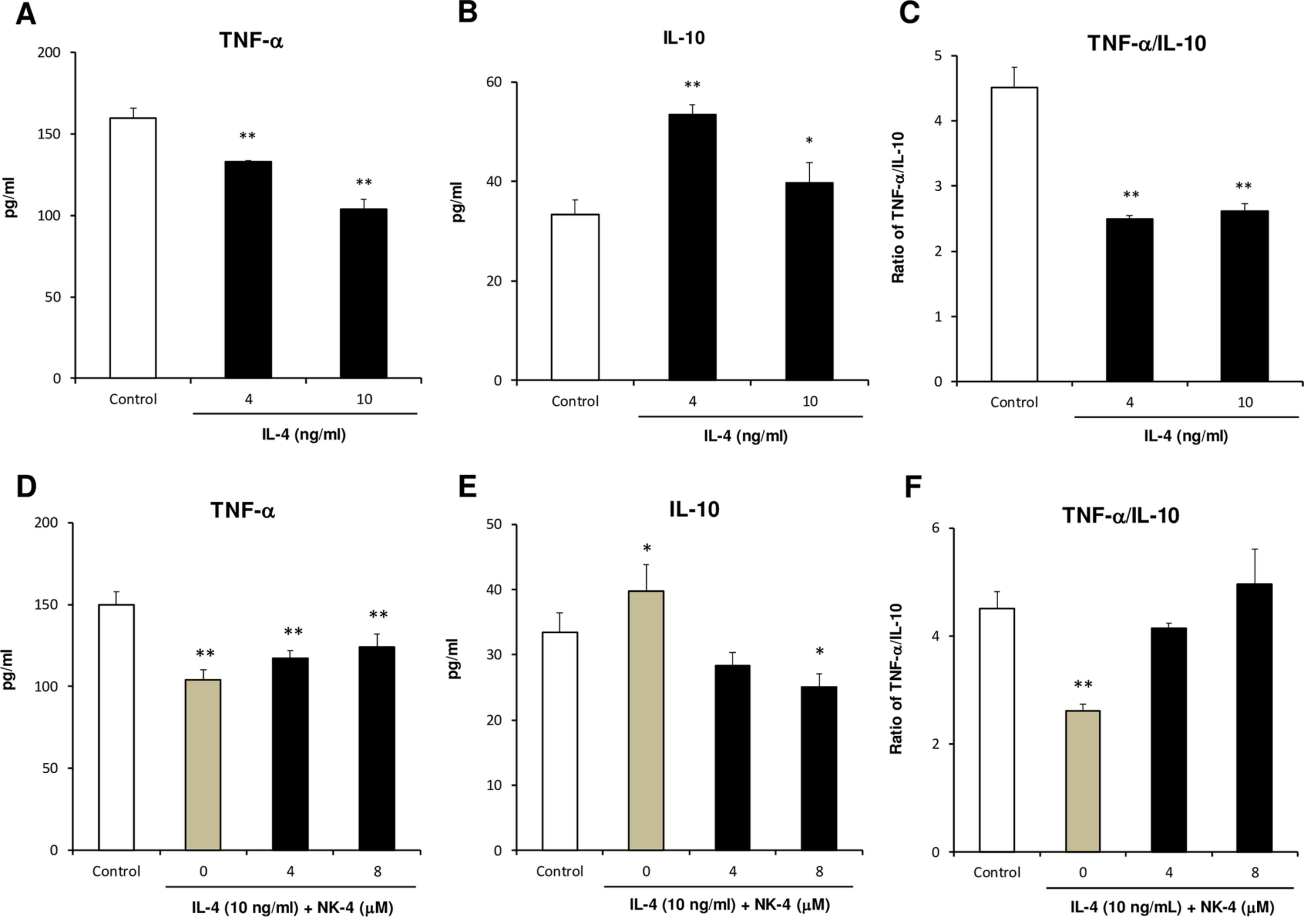
NK-4 inhibits the IL-4-driven alteration of cytokine expression profile in THP-1 cells. THP-1 cells were cultured with 4 ng/ml and 10 ng/ml IL-4 for 3 days at 37°C in RPMI1640 medium containing 1% FCS in 12-well culture plate (A to C). In a separate experiment, THP-1 cells were cultured with 10 ng/ml IL-4 in the presence or absence of varying concentrations of NK-4 (D to F). After pre-treatment with IL-4, THP-1 cells (1.2 x 10^5^ cells/well) in fresh RPMI1640 medium containing 10% FCS were stimulated overnight with LPS and the levels of TNF-α and IL-10 in the culture supernatants were determined by specific ELISA. The ratios of TNF-α to IL-10 are shown (C and F). Results are the means ± S.D. of triplicate cultures. Results are representative of three independent experiments with similar results. *p < 0.05, **p < 0.01 compared with control cultures.

Reflecting functional diversity and plasticity, macrophages polarize largely into two phenotypes, M1 and M2, depending on exposure conditions. M1 macrophages (classically activated macrophages) are derived by exposure to IFN-γ and/or LPS, and express predominantly pro-inflammatory cytokines, such as TNF-α and IL-1β [23]. M2 macrophages are derived by exposure to the Th2 cytokines IL-4 and IL-13, and produce anti-inflammatory cytokine such as IL-10 [23, 24]. M2 macrophages polarized by exposure to IL-4 or IL-13 are also called alternatively activated macrophages (AAM). Increased numbers of AAM were detected in asthmatic patients, and their numbers correlated with the severity of asthma [25]. Phorbol 12-myristate 13-acetate (PMA)-differentiated THP-1 cells were shown to polarize into IL-10-producing AAM-like macrophages by incubating them with IL-4 and IL-13 for 72 h [26]. In this study, we examined whether IL-4 could modulate cytokine production profiles of undifferentiated THP-1 cells. We found that when THP-1 cells are stimulated with LPS after pre-incubation with IL-4 for 72 h, IL-10 production was significantly up-regulated and TNF-α production was inversely and significantly down-regulated (Fig 8A and 8B). The ratio of TNF-α to IL-10 in IL-4-treated THP-1 cells was therefore decreased to 55% to 58% of the control culture (Fig 8C). These results suggest that IL-4 could modulate cytokine production profile of THP-1 cells from proinflammatory to anti-inflammatory responses. Using this experimental system, we assessed the effect of NK-4 on IL-4-induced alteration of cytokine expression. When THP-1 cells pre-treated with 10 ng/ml IL-4 for 72 h in the presence of NK-4 were stimulated with LPS, TNF-α production was increased and IL-10 production was inversely decreased in an NK-4-dependent manner, compared with THP-1 cells pre-treated with IL-4 alone (Fig 8D and 8E). The TNF-α/IL-10 ratio in THP-1 cells pre-treated with 10 ng/ml IL-4 and 8 μM NK-4 recovered to that of control culture (Fig 8F). These results indicate that NK-4 inhibits the IL-4-induced alteration of cytokine expression profile in THP-1 cells.

### NK-4 suppresses the STAT6 signaling pathway in NHDF stimulated with IL-4 and TNF-α

**Fig 9:**
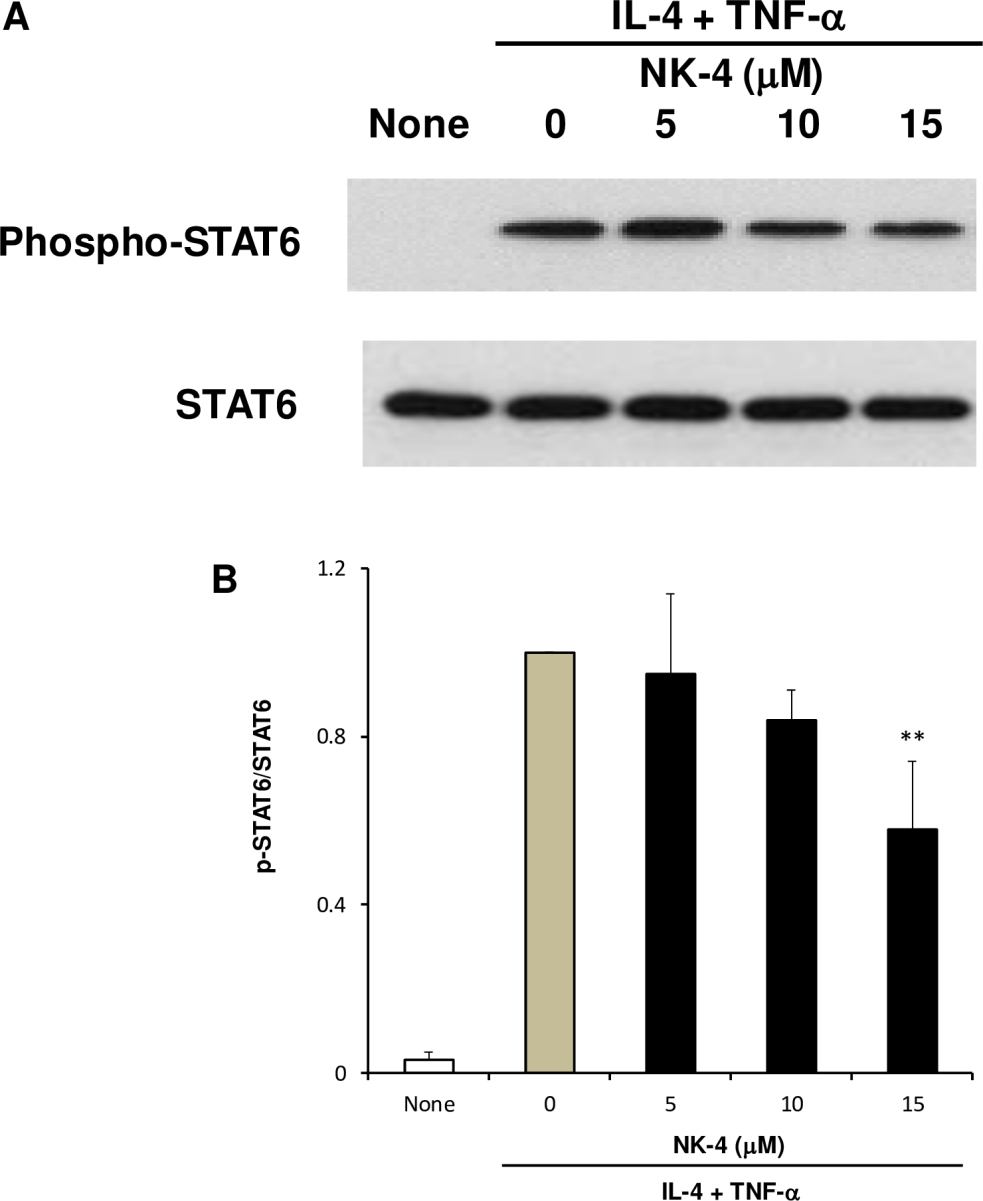
NK-4 suppresses the STAT6 signaling pathway in NHDF stimulated with IL-4 and TNF-α. NHDF were grown in confluent monolayer cultures in 12-well plates and were stimulated with 10 ng/ml IL-4 and 5 ng/ml TNF-α in the presence or absence of varying concentrations of NK-4 for 15 min. Phosphorylation of STAT6 was determined by immunoblotting whole-cell lysates using specific antibodies against the phosphorylated or total STAT6 protein. A representative blot is shown (A). The optical density ratio of phospho-STAT6 to total STAT6 is shown (B). Data from three independent experiments were combined and expressed as the means ± SD. **p < 0.01 compared with control cultures. Original uncropped and unadjusted Western blots were provided as supplementary files (S1 Fig and S2 Fig).

IL-4 and IL-13 bind to the type II IL-4 receptor, which is composed of IL-4Rα and IL-13Rα1 subunits and is mainly expressed by cells of nonhemopoietic origin, such as epithelial cells and fibroblasts, and activate the cell signaling molecule STAT6. The STAT6 signaling pathway has been shown to be critical for both IL-4- and TNF-α-induced eotaxin production by human dermal fibroblasts [18]. In cells unable to express STAT6, eotaxin was not detected after stimulation with either or both IL-4 and TNF-α. In our study, we showed that NK-4 decreased IL-4- and/or TNF-α-stimulated secretion of eotaxin and TARC by NHDF and inhibited the IL-4-driven alteration of cytokine expression profile in THP-1 cells. These results suggest that NK-4 would suppress the IL-4/STAT6 signaling pathway. To assess this possibility, we examined the effect of NK-4 on the phosphorylation of STAT6 in NHDF stimulated with IL-4 and TNF-α by Western blotting. As shown in Fig 9A, STAT6 was phosphorylated when NHDF were stimulated with 10 ng/ml IL-4 and 5 ng/ml TNF-α. This phosphorylation was suppressed by the addition of NK-4 in a concentration-dependent manner, whereas NK-4 had no effect on total STAT6 protein (Fig 9A and 9B). These results suggest that NK-4 decreased the production of eotaxin and TARC, at least in part, through inhibiting the STAT6 signaling pathway that is activated by IL-4 and TNF-α.

## Discussion

The active ingredient of the antiallergic drug Lumin is called cryptocyanine O.A. complex, which consists of three components, cryptocyanine O.A.1, cryptocyanine O.A.2, and cryptocyanine O.A.3. NK-4 is the common name of cryptocyanine O.A.1 and is contained more than 98% in the cryptocyanine O.A. complex. Cryptocyanine O.A. complex was first synthesized as cyanine photosensitizing dye by Dr. Kitaro Ogata in 1924. In 1940s, various studies were carried out for the exploration of its potential role in clinical applications. Among them, cryptocyanine O.A. complex was discovered to have immunomodulatory activities to treat allergic diseases and to promote wound healing. Cryptocyanine O.A. complex exhibited antihistaminic action in guinea pig. Furthermore, oral application of cryptocyanine O.A. complex (0.1 mg/body/day for 20 days) to the asthmatic patients resulted in the alleviation of asthmatic attack. In another study, alleviation or discontinuation of asthmatic attack was also observed in 7 out of 9 patients by intravenous administration of cryptocyanine O.A. complex at 1.5 mg/body to 7.75 mg/body every day for 3 to 15 days. Based on these preclinical and clinical data, Lumin has been in popular usage for the treatment of allergic diseases since 1951. Lumin is now available as a third-class over-the-counter drug in Japan.

In this study, we examined whether NK-4 exerts a regulatory effect on the activation and effector function of Th2 cells. NK-4 selectively inhibited the secretion of Th2 cytokines by primary splenic T cells as well as by Th2 cells. Particularly, the secretion of IL-4 and IL-5 by immobilized anti-CD3ε mAb-stimulated D10.G4.1 was abrogated by NK-4, suggesting that NK-4 directly acts on Th2 cells. Mechanistic analyses showed that NK-4 down-regulated GATA-3 and NFATc1 mRNA expression in anti-CD3ε mAb-activated Th2 cells.

GATA-3 is a master regulator of Th2 differentiation and function and plays a critical role in the differentiation of Th2 cells from naïve CD4^+^ cells. When mouse conventional T cells infected with a GATA-3-expressing retrovirus were stimulated with anti-CD3 Abs, the expression of IL-4, IL-5 and IL-13 were strongly increased [27]. GATA-3 also plays important roles in the maintenance of Th2 cytokine production in differentiated Th2 cells [28]. Furthermore, GATA-3 plays a pivotal role in type 2 cytokine production by group 2 innate lymphoid cells (ILC2) [29]. ILC2 are a recently identified cell population that are involved in the development of allergic inflammation as well as in parasite expulsion by producing the type 2 cytokines IL-5 and IL-13 in response to IL-25, IL-33, and thymic stromal lymphopoietin, which are secreted from epithelial cells after parasite or allergen exposure [30]. For these reasons, a recently developed therapeutic strategy to cleave and inactivate GATA-3 was studied in allergic asthmatic patients with promising results [31].

The NFAT family member NFATc1 (also called NFAT2) plays a role in IL-4 production by T cells. Overexpression of NFATc1 in mouse conventional T cells using retroviral infection resulted in increased IL-4 production, although not as strongly as GATA-3 [27]. T cells from mice lacking NFATc1 in the lymphoid system had reduced IL-4 production, whereas IFN-γ production was not affected [32]. Furthermore, it has been shown that NFATc1 in concert with GATA-3 or c-Maf drives IL-4 gene transcription [33–35]. In this regard, Yagi R et al. reported that expression levels of NFATc1 and GATA-3 are important factors that affect the potential of naïve T cells to secrete IL-4 [36]. Therefore, it seems likely that NK-4 inhibited IL-4 production by anti-CD3ε mAb-stimulated splenic primary T cells by down-regulating GATA-3 and NFATc1 mRNA expression.

Examining the effect of NK-4 on T-bet mRNA expression in Th1 cells would be helpful for supporting the selective regulation of Th2 activation by NK-4. T-bet is involved in the commitment of T helper cells to the Th1 lineage through direct activation of IFN-γ gene transcription [37]. T-bet deficiency impairs IFN-γ production in Th1 cells as well as CD4^+^ T cells [37, 38]. Furthermore, IFN-γ inversely enhances T-bet expression in Th1 cells [39].

In our study, however, T-bet mRNA expression was not examined in Fig 5, since T-bet is not expressed in D10.G4.1 cells [37]. NK-4 had little effect on IFN-γ production by anti-CD3ε mAb-stimulated BALB/c mouse spleen cells. Furthermore, IFN-γ secretion by Th1 cells in response to immobilized ant-CD3ε mAb was unchanged. These results suggest that NK-4 has little effect on T-bet expression. In addition, it seems unlikely that NK-4 inhibited GATA-3 mRNA expression through up-regulation of T-bet expression, although it is recognized that T-bet suppresses the expression and function of GATA-3 [40]. For these reasons, we consider that examining the effect of NK-4 on T-bet mRNA expression is a subject of future investigation.

It has been demonstrated that there are two modes of GATA-3 expression in T cells [41]. In naïve CD4^+^ T cells, IL-4/STAT6 signaling is required for GATA-3 induction. In differentiated Th2 cells, TcR signaling induces GATA-3 expression without the need for IL-4/STAT6 signaling [41]. In this study, we showed that NK-4 down-regulated GATA-3 mRNA expression in immobilized anti-CD3ε mAb-stimulated D10.G4.1 cells, suggesting that down-regulation of GATA-3 mRNA by NK-4 may be independent of the IL-4/STAT6 signaling pathway. We demonstrated that NK-4 suppressed the STAT6 signaling pathway in type II IL-4 receptor-expressing NHDF cells when stimulated with IL-4 and TNF-α. But it remains unclear whether NK-4 inhibits the IL-4/STAT6 signaling pathway in naïve CD4^+^ T cells, which primarily express the type I IL-4 receptor composed of IL-4Rα and common γ chain. The effect of NK-4 on IL-4-induced differentiation of naïve CD4^+^ T cells to Th2 cells remains to be clarified. However, our findings that NK-4 inhibited IL-4 and IL-5 secretion by Th2 cells through down-regulating GATA-3 and NFATc1 mRNA expression suggest that NK-4 could be an effective strategy for the treatment of Th2-mediated allergic disease.

The concentrations at which NK-4 inhibited mRNA expression of GATA-3 or NFATc1 were higher than those at which NK-4 inhibited IL-4 and IL-5 production by antigen-stimulated D10.G4.1 cells. We consider that this was due to the difference in the stimulatory conditions. The effects of NK-4 on GATA-3 and NFATc1 mRNA expression were examined with D10.G4.1 cells stimulated with immobilized anti-CD3ε mAb, where higher concentrations of NK-4 were needed to cause significant inhibition of IL-4 and IL-5 secretion (Fig 3) compared with those observed with antigen stimulation (Fig 4).

It is well recognized that the immobilized anti-CD3 mAb delivers strong stimulatory signals compared with stimulation by antigen or soluble anti-CD3 mAb in the presence of accessory cells [42]. In fact, when D10.G4.1 cells were stimulated with immobilized anti-CD3ε mAb, 20.5 ng/ml IL-4 and 4.8 ng/ml IL-5 were secreted in the control cultures (Fig 3), whereas 2.4 ng/ml IL-4 and 3.0 ng/ml IL-5 were produced by antigen stimulation (Fig 4). Similar findings were observed with Th1 clone #4 cells.

It is possible that the differences in the strength of stimulatory signals through TcR-CD3 complex between antigen stimulation and the immobilized anti-CD3ε mAb stimulation affected the effective doses of NK-4. For this reason, we consider that higher concentrations of NK-4 were necessary to cause significant inhibition of both GATA-3 and NFATc1 mRNA expression and Th2 cytokine secretion by D10.G4.1 cells in response to immobilized anti-CD3ε mAb.

Persistent inflammation accompanied by prominent infiltration of eosinophils is a pathological hallmark of allergic asthma, probably due to activated oxygen species, toxic granular proteins and lipid mediators released from activated eosinophils [43]. Infiltration of eosinophils was also observed in the skin lesions of atopic dermatitis patients [44]. Eotaxin is a potent chemoattractant and activator of eosinophils and basophils. Enhanced expression of eotaxin is observed in the asthmatic lung as well as in lesional skin from atopic dermatitis patients [6, 16]. Thus, we investigated the regulatory effect of NK-4 on Th2 cytokine-induced chemokine secretion (eotaxin and TARC) by dermal fibroblasts to model the effector phase of type 2 immune responses. Our results showed that NK-4 inhibited eotaxin secretion by NHDF cells stimulated with IL-4 and/or TNF-α.

In addition to the abrogation of eotaxin production, NK-4 significantly and dose-dependently inhibited the production of TARC by NHDF cells stimulated with NK-4 and TNF-α. Interestingly, FK-506 also inhibited TARC production at concentrations over 5 μM, which corresponded to more than 500 times higher than those reported for inhibiting cytokines secretion by antigen-stimulated T cells [45]. This inhibition was not due to cytotoxicity to NHDF, since FK-506 had little effect on the cell number of NHDF at the concentrations used (data not shown). FK-506 is commonly used as an immunosuppressant (Tacrolimus ointment) for the treatment of atopic dermatitis. Immunosuppressive effects of FK-506 on T cells, mast cells and keratinocytes are well studied [46]. Our findings that FK-506 inhibited TARC production by IL-4/TNF-α-stimulated NHDF may add further insight into therapeutic mechanism of action of this drug, although the mechanism of inhibition needs to be addressed.

Dexamethasone did not inhibit TARC production, even though concentrations were increased to 15 μM; instead, increased TARC production was observed with dexamethasone over the range of concentrations from 0.03 μM (data not shown) to 15 μM (Fig 7A). Staples KJ reported that TARC production by IL-4-treated human monocyte-derived macrophages is corticosteroid- resistant [47]. These results suggest that TARC production may be insensitive to corticosteroid treatment, at least in macrophages and fibroblasts. These results further highlight the clinical significance of NK-4 for the treatment of steroid-resistant allergic inflammatory diseases.

Serum TARC levels are well correlated with clinical scores for atopic dermatitis, and TARC has been used as clinical biomarker for the treatment of atopic dermatitis [48], suggesting the importance of this chemokine in the pathogenesis of atopic dermatitis. Our results suggest that NK-4 may prevent tissue damage and inflammation observed in allergic diseases by inhibiting the recruitment of eosinophils and Th2 cells through down-regulating the secretion of eotaxin and TARC by epithelial cells and fibroblasts in the lung and dermis in response to IL-4 and TNF-α. Since both IL-4 and TNF-α are necessary to induce TARC expression in NHDF, we believe that inhibition of the STAT6 signaling pathway by NK-4 is responsible, at least in part, for the down-regulation of Th2 chemokine secretion.

The effective concentrations of NK-4 to inhibit STAT6 phosphorylation in NHDF were higher than those required to inhibit TARC production. Shoda T et al. showed that activation of both NF-κB and p38MAPK was involved in the TARC expression by dermal microvascular endothelial cells, since inhibitors for NF-κB and p38MAPK decreased TARC secretion [20]. These results together with our findings suggest that at least three signal transduction molecules, STAT6 activated by IL-4, and NF-κB and p38MAPK, both of which are probably activated by TNF-α, are involved in the TARC expression.

In our preliminary study, NK-4 inhibited TNF-α secretion by mouse peritoneal macrophages stimulated with both LPS and IFN-γ, suggesting that NK-4 may exhibit anti-inflammatory effect under certain conditions by inhibiting NF-κB or MAPK signaling pathways. This further suggests a possibility that NK-4 down-regulated TARC secretion from IL-4/TNF-α-stimulated NHDF by regulating signaling events involving NF-κB or MAPK activation as well as STAT6 activation. For these reasons, we assume that NK-4 inhibited TARC secretion at lower concentrations than those required to inhibit STAT6 phosphorylation. Further studies should be necessary to attest this possibility.

Similar to that observed with NK-4, it has been shown that suplatast tosilate (IPD-1151T), an antiallergic drug that selectively inhibits Th2 cytokine production, down-regulates Th2 chemokine secretion by human dermal fibroblasts and Th2 cells [22, 49]. We compared the down-regulatory effect of suplatast tosilate on TARC production with that of NK-4. Our results indicate that suplatast tosilate was qualitatively similar but had quantitatively attenuated inhibitory activity compared with that of NK-4. This suggests that NK-4 is an attractive alternative Th2-selective antiallergic agent.

In this study, we showed that IL-4 could modulate cytokine production profile of THP-1 cells from proinflammatory to anti-inflammatory response (down-regulation of TNF-α and up-regulation of IL-10), when THP-1 cells were pre-treated with IL-4 for 3 days before LPS stimulation. This may reflect the anti-inflammatory properties of IL-4. As anti-inflammatory cytokine, it has been shown that IL-4 inhibits the production of TNF-α and IL-12, and enhances IL-10 production by LPS-stimulated macrophages [50, 51]. Pre-treatment of THP-1 cells with IL-4 and NK-4 resulted in inhibition of IL-4-driven alteration of cytokine expression profile, suggesting that NK-4 is able to down-regulate IL-4-mediated signaling events, even in macrophages. These are important findings that could lead to prevention of IL-4-induced polarization to AAM. Development of macrophages with an M2 (AAM)-like phenotype has been proposed in patients with asthma [25, 47]. Sputum macrophages expressing significantly increased levels of TARC mRNA, as observed in IL-4-treated monocyte-derived macrophages, was reported in asthmatic patients [47]. Research is starting to address the effect of NK-4 on IL-4-induced polarization to AAM phenotype using THP-1 cells morphologically and functionally according to the accepted protocol [26].

Recommended clinical doses of NK-4 for the treatment of allergic disease are 50 to 100 μg/body. In fact, oral application of cryptocyanine O.A. complex (0.1 mg/body/day for 20 days) to the asthmatic patients resulted in the alleviation of asthmatic attack as mentioned earlier. These effective concentrations of NK-4 in the clinical studies were substantially lower than those required to cause functional changes in Th2 cells and NHDF *in vitro* such as inhibition of Th2 cytokine and chemokine secretion as shown in this study (2 μM to 15 μM NK-4, corresponding to 0.4 μg to 3 μg of NK-4 per well in 96-well plates). At present, it remains to be determined why low doses of NK-4 alleviate clinical signs of allergic diseases.

Previously we showed that when graded doses (1, 10, 100 and 1,000 μg/kg) of NK-4 were administered orally to C57BL/6N mice once daily for 3 days, IFN-γ secretion by LPS-stimulated spleen cells from these mice significantly increased in a dose-dependent manner and reached a plateau level (5.2-fold of control mice) at 10 μg/kg NK-4. On the other hand, there was no detectable difference in IL-6 secretion regardless of the doses of NK-4 administered [52]. Significant increase in IFN-γ secretion (3.1-fold of control mice) was also observed even at 1 μg/kg NK-4. We demonstrated that IL-12 and NKT cells were involved in this IFN-γ secretion [9]. These results suggest that low concentrations of NK-4, which was given orally, could be delivered systemically, leading to activation of IFN-γ-producing cells in the spleen.

It is well recognized that IFN-γ counteracts Th2 immune responses by reducing Th2 cytokine expression and suppressing the development of Th2 cells [53]. Therefore, it seems that IFN-γ induced by NK-4 *in vivo* may lower the effective doses of NK-4 in concert with direct regulatory effect of NK-4 on the activation and effector function of Th2 cells as shown in this study. Further studies should be necessary to attest this possibility.

## Conclusions

We demonstrated that NK-4 exhibited selective down-regulation of Th2 cytokine production by antigen- or anti-CD3ε mAb-activated Th2 cells. In addition, NK-4 inhibited the secretion of eotaxin and TARC by IL-4/TNF-α-activated fibroblasts, both of which are important Th2 chemokines responsible for the infiltration of eosinophils and Th2 cells during the effector phase of allergic inflammation. Furthermore, NK-4 abrogated the IL-4-driven alteration of cytokine expression profile in THP-1 cells, suggesting a regulatory effect of NK-4 on IL-4-mediated signaling events that are involved in polarization to AAM phenotype, which is proposed to have pathogenic roles in allergic asthma. The importance of Th2 cells in the development and progression of type 2 inflammatory disorders has been increasingly highlighted. Our results provide a rationale for the use of NK-4 as a therapeutic agent for Th2-mediated allergic inflammation.

## Supporting information

S1 Fig Original uncropped and unadjusted Western blot of phosphorylated STAT6 protein. Original uncropped and unadjusted Western blot of phosphorylated STAT6 protein was presented as supporting information of Fig 9.
(TIF)

S2 Fig Original uncropped and unadjusted Western blot of total STAT6 protein. Original uncropped and unadjusted Western blot of total STAT6 protein was presented as supporting information of Fig 9.
(TIF)
