# Supplementary figures and images for: NK-4 exerts selective regulatory effects on the activation and function of allergy-related Th2 cells
Source: PLoS One. 2018 Jun 22;13(6):e0199666. doi: 10.1371/journal.pone.0199666 (PMC6014662; doi:10.1371/journal.pone.0199666)

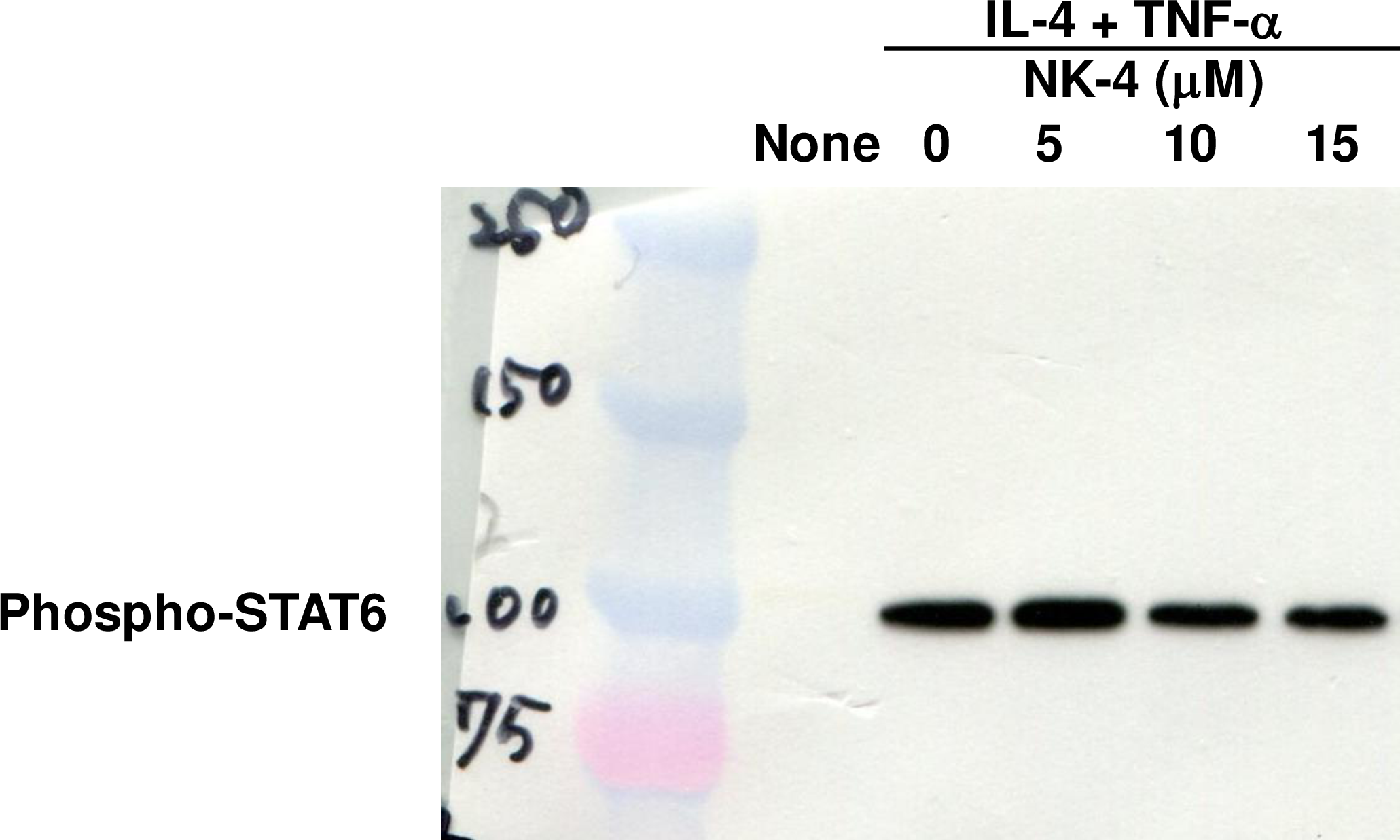

Supplement: S1 Fig — Original uncropped and unadjusted Western blot of phosphorylated STAT6 protein was presented as supporting information of Fig 9. (TIF) [file pone.0199666.s001.tif]

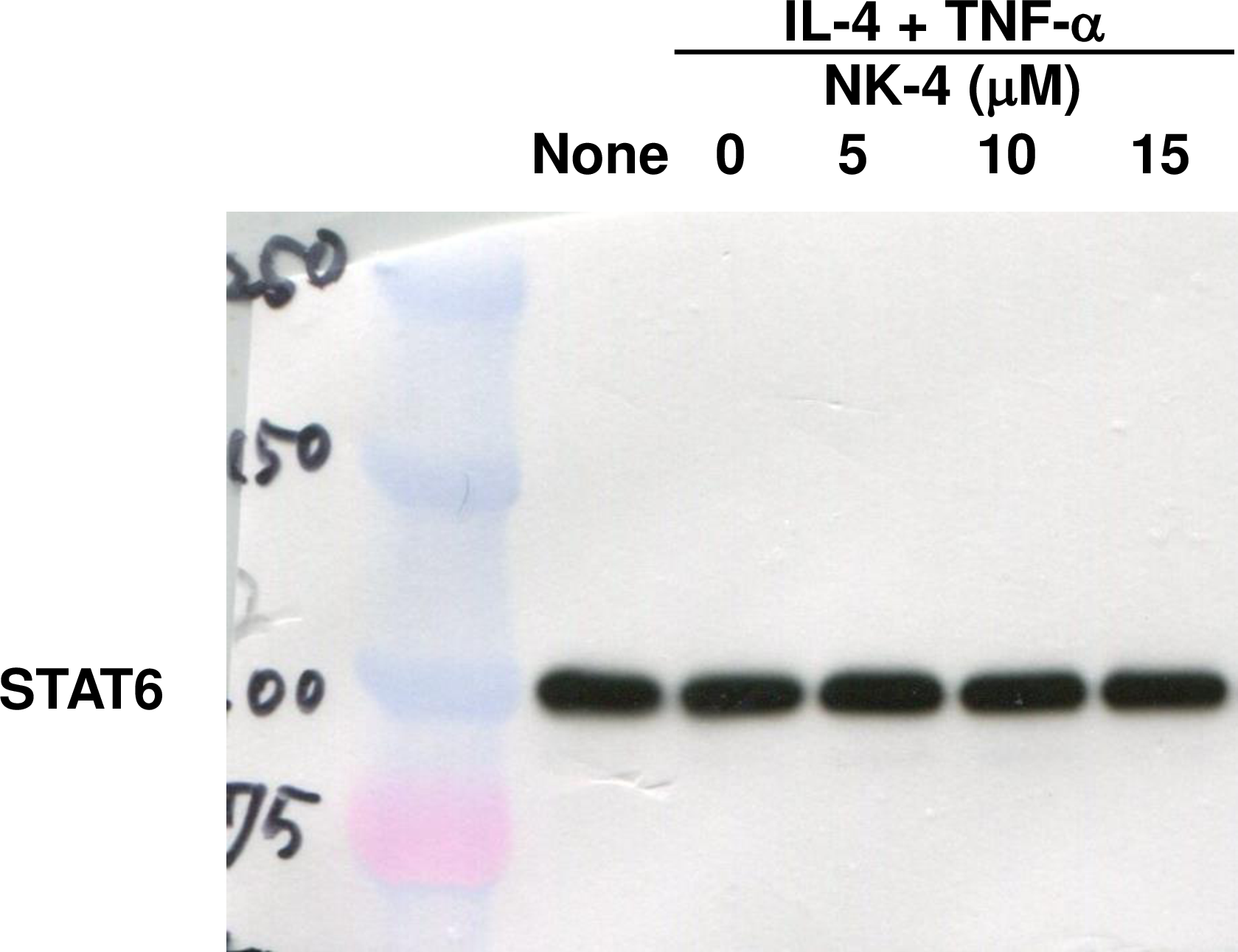

Supplement: S2 Fig — Original uncropped and unadjusted Western blot of total STAT6 protein was presented as supporting information of Fig 9. (TIF) [file pone.0199666.s002.tif]
